# The Predictive Validity of Item Effect Variables in the *Satisfaction With Life Scale* for Psychological and Physical Health

**DOI:** 10.1177/10731911221149949

**Published:** 2023-02-08

**Authors:** Marie-Ann Sengewald, Tina H. Erhardt, Timo Gnambs

**Affiliations:** 1 Leibniz Institute for Educational Trajectories (LIfBi), Bamberg, Germany; 2 Otto-Friedrich-University Bamberg, Germany

**Keywords:** life satisfaction, latent state-trait theory, measurement error, item effect, validity

## Abstract

Although the *Satisfaction with Life Scale* strives to capture a single dimension, describing respondents’ satisfaction with life as a whole, individual items might also capture unique aspects of life satisfaction leading to some form of multidimensionality. Such systematic item-specific variance can be viewed as a content-laden secondary trait. Information on the nomological net and predictive validity can be useful to aid the interpretation of these item-specific effects. Therefore, the present study on *N* = 2,543 Dutch respondents adopts revised latent state-trait theory to disentangle common construct variance, random measurement error, and person-specific item effects in the *Satisfaction with Life Scale* across three measurement occasions. The reported analyses not only demonstrate how to examine item-specific multidimensionality in longitudinal data but also emphasize how different identification constraints for the latent variable lead to different interpretations. Moreover, the predictive validity of item effect variables for the prediction of psychological and physical health is examined. A cross-validation with the same sample at a later measurement period and robustness checks with incomplete data, support our findings on the substantive value of a multidimensional specification of the *Satisfaction with Life Scale* for substantive analyses. Finally, the contributions of person-specific item effects for psychological assessments are discussed.

A plethora of psychometric research highlighted various sources of systematic variation that can affect multi-item measurements next to the latent attribute a scale intends to measure (e.g., Meredith, 1993; Millsap, 1997, 2007; Podsakoff et al., 2003; van Bork et al., 2022). Even when different items aim to measure the same attribute, semantic multidimensionality or wording effects may occur due to differences in the item formulations (e.g., Gnambs, 2015; Gu et al., 2017; Marsh et al., 2010; Ponce et al., 2021; Schroeders & Gnambs, 2020). Furthermore, the raters’ familiarity with the item content or individual response styles can introduce systematic variation in measurements (e.g., Adams et al., 2019; Liu et al., 2017). Recent research also highlighted that even single items can carry substantial meaning beyond the common trait (e.g., Achaa-Amankwaa et al., 2021; McCrae et al., 2019; Stewart et al., 2021), which might manifest as multidimensionality for individual items in longitudinal data based on the assumption of stable individual item effects across multiple measurement occasions (e.g., Eid, 1996; Geiser & Lockhart, 2012; Kenny, 2021; Marsh & Grayson, 1994).

Following the (revised) latent-state-trait (LST-R) theory (Steyer et al., 2015), the response of a person to an item is determined by four factors, (a) the attribute of the person at the occasion of measurement typically referred to as a trait, (b) the situation in which the person is assessed, (c) measurement error (random variation), and (d) systematic effects of an item. To account for the latter, previous examinations of psychological measures have acknowledged item-specific traits (e.g., Eid & Kutscher, 2014; Joshanloo, 2022; López-Benítez et al., 2019; Scarpato et al., 2021), or method effects for individual items (Cogo-Moreira et al., 2021; Erhardt et al., 2022; Geiser et al., 2019; Holtmann et al., 2020; Thielemann et al., 2017). Although modeling item-specific traits allows disentangling situation-specific effects and modeling a latent trait for each item across different time points, this approach confounds common and specific item effects (i.e., Factors *a* and *d*). Instead, method effects separate the item-specificity (Factor *d*) from effects that are common for all items but are situation specific (i.e., states that include Factors *a* and *b*), offering the possibility for further investigations on person-specific item effects themselves. So far, little is known whether person-specific item effects represent mere nuisance fluctuation introducing bias in measurements, or, rather, they carry substantial meaning beyond the focal construct.

The present study demonstrates the potential of person-specific item effects for providing a nuanced specification of a latent attribute of interest and exemplifies this approach with the five-item *Satisfaction with Life Scale* (SWLS; Diener et al., 1985). For this, we first describe the measurement of life satisfaction with the SWLS and highlight its problematic dimensionality. Then, we discuss how person-specific item effects can contribute useful information for substantial analyses. In our application, we demonstrate the importance of acknowledging such effects in the SWLS and investigate their contribution for predicting indices of psychological and physical health.

## The (Multi-)Dimensionality of the SWLS

The SWLS (Diener et al., 1985) is a brief instrument including only five items for measuring the cognitive aspect of subjective well-being in the form of life satisfaction ratings (see Table 1 for the items). In contrast to other instruments that also consider life satisfaction in specific domains such as relationships, health, or finances, for example, the *Freiburg Personality Inventory* (FPI-R; Fahrenberg et al., 1984), the SWLS aims to capture the respondents’ satisfaction with life as a whole. Thus, the basic idea is that the respondents use their own criteria to make judgments of their global life satisfaction by incorporating and weighting different domains (cf. Diener et al., 1985; Pavot & Diener, 1993). Yet, when respondents assess the global quality of their lives, it is unclear whether the individual items might also capture unique aspects of life satisfaction leading to some form of multidimensionality.

**Table 1.** Satisfaction With Life Scale (SWLS; Diener et al., 1985).

Below are five statements with which you may agree or disagree. Using the 1–7 scale below, indicate your agreement with each item by placing the appropriate number on the line preceding that item. Please be open and honest in your responding.	
1	In most ways, my life is close to my ideal
2	The conditions of my life are excellent
3	I am satisfied with my life
4	So far I have gotten the important things I want in life
5	If I could live my life over, I would change almost nothing

*Note.* Response scale: 1 = strongly disagree, 2 = disagree, 3 = slightly disagree, 4 = neither agree nor disagree, 5 = slightly agree, 6 = agree, 7 = strongly agree.

The development of the SWLS was guided by a unidimensional conceptualization and supported a single dimension using principal-axis factor analysis (Diener et al., 1985). Subsequently, the SWLS has been repeatedly subjected to exploratory and confirmatory factor analytic research (see Pavot & Diener, 2008, for a review). Although these studies overwhelmingly demonstrated that a single factor tends to account for the majority of the variance of the responses to the SWLS, some analyses indicated that different items might exhibit also unique variance (see Erhardt et al., 2022; Holtmann et al., 2020; Pavot & Diener, 1993, 2008). Especially, the fifth item of the scale (“If I could live my life over, I would change almost nothing”) frequently showed lower factor loadings and item-total correlations as compared with the first four items of the scale (e.g., Pavot & Diener, 2008; Senecal et al., 2000). Some research even suggested that the items might capture different facets of life satisfaction because the last two items refer to the past, whereas the remaining items address current satisfaction (e.g., Bai et al., 2011; Hultell & Gustavsson, 2008; Sachs, 2003).

Thus, although the SWLS is typically considered a unidimensional scale, there is some evidence that systematic differences exist between the items. With regard to the construction of the scale—building on the respondent’s individual cognitive processes—it is plausible that the five items differ not only by a constant difficulty or discrimination parameter, but differences for assessing life satisfaction with different items can be person-specific. This is supported by recent applications of Erhardt et al. (2022) and Holtmann et al. (2020), who modeled SWLS items with person-specific item effects. So far, these types of analyses are rare in applied research, because they have rather strong data requirements and involve complex psychometric models. An introduction into one of these modeling approaches that allows for the specification of person-specific item effects in longitudinal data is provided in the Appendix. These challenges notwithstanding, psychometric analyses with item-effect variables can help gain a better understanding of the substantive contribution of person-specific item effects to explaining psychological or behavioral outcomes of subjective well-being.

## The Contribution of Person-Specific Item Effects for Subsequent Analysis

Previous research on person-specific item effects highlighted several advantages of the more complex psychometric models in comparison to unidimensional construct definitions at each time point (Cogo-Moreira et al., 2021; Erhardt et al., 2022; Geiser et al., 2019; Holtmann et al., 2020; Thielemann et al., 2017) such as (a) less restrictive assumptions on the factorial structure, which can substantially increase model fit, (b) a more accurate specification of the latent states that account for person-specific item effects in the response process, or (c) the possibility to study the person-specific item effects, for instance, in test construction (e.g., for item selection). However, so far, the contribution of person-specific item effects for applied psychological research has received little attention. Therefore, we consider different perspectives and further investigations on the meaning of person-specific item effects in the SWLS.

## Different Perspectives on the Meaning of Person-Specific Item Effects

The presence of person-specific item effects in the SWLS suggests that different items for assessing life satisfaction are understood or dealt with differently by the respondents. Such effects have also been referred to as “systematic error” (see van Bork, 2019; van Bork et al., 2022) which implies two opposite interpretational perspectives: The person-specific item effects are systematic and (a) contribute useful, content-related information for substantial analyses or (b) reflect a form of measurement error, thus, representing nuisance for substantive analyses.

Following the first perspective, person-specific item effects can be considered stable person characteristics that measure a more nuanced concept of life satisfaction based on the item content. Like in individual difference research focusing on so-called personality nuances (e.g., McCrae et al., 2019; Stewart et al., 2021), where item effects are considered secondary traits that are closely related to the focal construct but reflect unique domain content not shared with the other items. Such a unique domain content for the SWLS items may refer to the different perspectives on life satisfaction. For example, Item 3 assesses life satisfaction most directly, while Items 1 and 2 are more specific as they refer to ideal and excellent life conditions. In contrast, Items 4 and 5 include a retrospective component that is not shared by the other items (see Bai et al., 2011; Hultell & Gustavsson, 2008; Sachs, 2003). Thus, one could imagine that a respondent might experience high life satisfaction, even though his or her conditions do not perfectly match one’s ideal. Even though someone would like to change parts of her/his current life, this might not strongly affect the global assessment of her/his life. As such, each item might capture a slightly different aspect of life satisfaction that is not shared by the other items and, thus, can reflect stable interindividual differences between persons beyond the common trait (i.e., global life satisfaction).

In contrast, the second perspective considers person-specific item effects as stable person characteristics, which are not conceptually related to life satisfaction, but represent distinct domain-independent effects. For instance, response styles of the persons can systematically affect item responses and will be captured as person-specific item effects, if they are not constant for different items, but interact with item characteristics like content, wording, or length (e.g., Adams et al., 2019; Kam & Fan, 2020; Liu et al., 2017). For the SWLS, the item instruction and response scale are equal for all items. Similarly, the items differ only slightly in length and complexity. Thus, structural differences between SWLS items may mainly refer to the specific content. Still domain-independent effects are possible, due to more general person characteristics like familiarity with the item content or motivation that can systematically affect the responses to (individual) items (van Bork et al., 2022). Also, other sources of method variance may be present, for instance, groups of persons can systematically respond differently to specific items. As an example, Holtmann et al. (2020) acknowledged rater-specific effects (i.e., differences between self, parent, and peer ratings), which can be disentangled from person-specific item effects in their modeling approach. An overview of different sources of method variance is provided in Podsakoff et al. (2003). Thus, while the second perspective treats person-specific item effects as a form of error that introduces bias in relations among latent constructs if it is not accounted for (e.g., Podsakoff et al., 2003), the first perspective views person-specific item effects as a facet of domain content that may be useful not only on psychometric grounds but also for substantive analyses. Although modeling person-specific item effects does not allow for distinguishing the different sources of multidimensionality, it allows for identifying whether multidimensionality is present and, more importantly, for further investigations on the item effects.

## Further Investigations on the Meaning of Person-Specific Item Effects

Scrutinizing person-specific item effects is important to gain a better understanding of the identified multidimensionality and can help discern different aspects of the focal construct. To do so, the specification of the latent variables becomes essential because the interpretation of the latent variables varies depending on how the latent variable was defined, that is, the chosen identification constraints (see the Appendix for psychometric details). Typically person-specific item effects are modeled as differences between a given item and a latent state variable as measured by a reference item. Consequently, the means, variances, and correlation coefficients of the latent state variables and item–effect variables can substantially vary depending on the chosen reference item. For example, choosing the third SWLS item as the reference, which measures life satisfaction most directly, will allow for investigating differences of all other items to this direct measure. Instead, when choosing the fifth SWLS item as the reference, which shows the largest differences in psychometric properties in the scale, will allow for describing differences to this retrospective evaluation of life satisfaction. From a methodological perspective, both identification constraints are equally valid and no preference can be given to either one. However, the choice matters from a substantive, content-related point of view because the resulting latent variables are interpreted differently. Thus, the choice of the reference item should be guided by theoretical considerations that allow properly addressing the specific research question at hand.

It is also straightforward to integrate a measurement model with person-specific item effects into a larger structural equation model to gain a deeper understanding of the response process. This has recently been demonstrated in the investigations of Erhardt et al. (2022) and Holtmann et al. (2020) for the SWLS and by Thielemann et al. (2017) for the life satisfaction scale of the FPI-R. For example, Holtmann et al. (2020) showed that person-specific item effects were robust across different rater groups, while Thielemann et al. (2017) examined several explanatory variables to explain item-effect variables. Finally, Erhardt et al. (2022) investigated person-specific item effects in a multi-construct context. They investigated the homogeneity of the correlation structure between item-effect variables and states, both within and between constructs. In their application, a heterogeneous correlation structure that matched the item content was considered as an indicator for semantic multidimensionality in the five items of the SWLS.

Although previous research demonstrated the robustness of person-specific item effects and also tried to explain them based on item content and bivariate relations with other constructs, little is known about their contribution to substantive analyses in terms of their incremental validity. Incremental validity investigates the degree to which a new measure of a construct explains or predicts a phenomenon of interest relative to other measures (e.g., Hunsley & Meyer, 2003). Accordingly, we are interested in whether person-specific item effects in the SWLS provide additional information for predicting relevant criterion variables beyond the common states. Our application focuses on measures of psychological and physical health because of the SWLS’ popularity in the epidemiological and clinical context (e.g., Pavot & Diener, 2008).

## The Present Study

The present study examines the relevance of person-specific item effects for predictive analyses. We apply a multi-state model with latent difference variables (e.g., Erhardt et al., 2022; see also Appendix) for the measurement of item effects in the SWLS (Diener et al., 1985) that was administered at three measurement occasions. As suggested by previous investigations (e.g., Erhardt et al., 2022), substantial person-specific item effects were expected that may be valuable for substantive analyses. Accordingly, we first detail the multidimensionality in our application and consider two different identification schemes. Then, we investigate the contribution of the item–effect variables for predictive analyses of two health outcomes (i.e., indices of psychological and physical health) and explore whether they explain incremental variance beyond the latent state variables. Finally, the generalizability of these results is demonstrated by replicating the analyses with the identic sample for different measurement periods.

## Method

### Sample and Procedure

The *Longitudinal Internet Studies for the Social Sciences* (LISS) panel follows a representative sample of the Dutch population since 2008 (Blom et al., 2016; Scherpenzeel & Das, 2010) by administering multiple web-based surveys on diverse topics such as personality or health each year. The panel is based on a probability sample of all households registered in the Netherlands. To achieve representativeness, respondents without computer or internet access are provided with the necessary technical equipment.

We report how we determined our sample size, all data exclusions, and all measures in the study. For the present analyses, we considered six measurement occasions from 2008 to 2013. The complete sample originally consisted of *N*_all_ *=* 10,133 respondents across all measurement occasions. However, we limited our sample to respondents that had at least one valid response on the focal instruments (see below) at each wave. Because the available sample at each measurement occasion varied substantially due to unit-nonresponse and sample refreshments, this resulted in *N*_t_ *=* 5,169 to 6,808 respondents depending on the wave. Listwise deletion was applied for participants that did not respond to at least one item at each measurement occasion. This resulted in an analysis sample of *N* = 2,543 respondents (51.86 % female) with an age range of 16 to 88 years (*M* = 50.25, *SD =* 14.91). About 29% of the analysis sample had higher vocational education such as college or university. For implementing a cross-validation, we divided the six measurement waves into two groups of three waves each, 2008 to 2010 and 2011 to 2013, respectively. All analyses were repeated twice for the *same respondents* but the different measurement periods. To guard against distortions resulting from our sample selection procedure, we also repeated the analyses on larger samples including respondents with only one valid response on the focal constructs at a *single* measurement occasion in either measurement period (incomplete Sample 1: *N*_p1_ *=* *5,549*, or 2: *N*_p2_ *=* *5,248*). This allowed us to investigate the generalizability of the results across non-response patterns. The results of these sensitivity analyses are reported in the Online Supplement.

### Instruments

The five SWLS items (Diener et al., 1985) were administered as part of a personality inventory on identical item positions (014–018) at all six measurement occasions. The items were presented in Dutch on 7-point response scale from 1 = strongly disagree to 7 = strongly agree. No missing values were observed for any item—meaning there was no item-specific non-response for the participants that responded on all six measurement occasions. The item means fell between 4.56 and 5.57, while the respective standard deviations ranged from 1.08 to 1.63 (see Table S1 in the Supplemental Material for descriptive statistics).

Health outcomes for the respondents were measured with two instruments in 2010 and 2013 (i.e., the last wave in each of the two analysis periods). The short *Mental Health Inventory* (MHI-5; de Moor et al., 2018; Ostroff et al., 1996) included five items measuring psychological distress, while the *Physical Health/Mobility Index* (PHI; Green & Young, 2001) comprised 23 items capturing problems with different activities (see Tables S2 and S3 in the Supplemental Material for the scale items). Four participants had missing values on single MHI-5 and PHI items. For both scales, we constructed an index by averaging the available item responses on the respective instrument for each person. The MHI-5 items were rated on six-point scales and coded in such a way that higher values indicated frequent distress. At both measurement occasions (2010 and 2013), the mean of this index fell around 2.20 with substantial variation (*SD* = 0.82). Because we examined a non-clinical sample, the distribution of the MHI-5 index was right-skewed with most participants reporting few mental health problems (see Table S4 in the Supplemental Material for descriptive statistics). The PHI items were rated on 5-point scales with higher values indicating more mobility problems. On average, the PHI scores fell around 1.20 at both measurement occasions, with slightly less variation in 2010 (*SD* = 0.34) as compared with 2013 (*SD* = 0.38). The distribution of the PHI index was right-skewed with most participants reporting few mobility problems (see Table S4 in the Supplemental Material for descriptive statistics).

### Statistical Analyses

**Figure 1.**
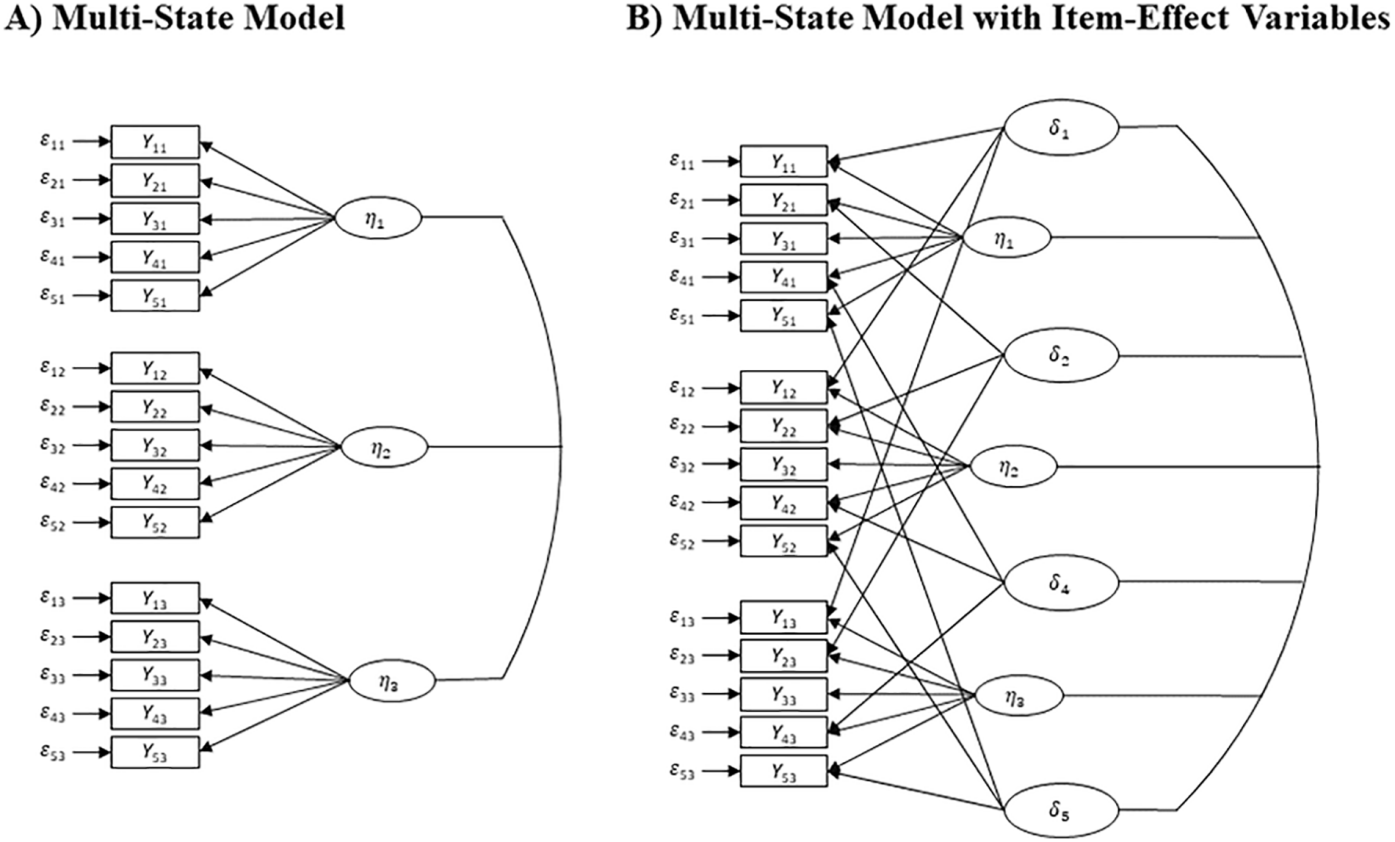
Multi-State Model With and Without Item–Effect Variables for the Satisfaction With Life Scale (Diener et al., 1985).

Multidimensionality in the SWLS was evaluated by comparing a multi-state model without item–effect variables to an extended model with item–effect variables (see Erhardt et al., 2022; Thielemann et al., 2017) as illustrated in Figure 1. The manifest variables *Y_it_* contain the responses to the *i* =1,…, 5 SWLS items for *t* =1 ,..., 3 time points. In the multi-state model without item–effect variables, we assumed *η_t_*-congenericity with strong measurement invariance for the latent state variables *η_t_*. In contrast, we constrained all intercepts and loadings to 0 and 1, respectively, for modeling item–effect variables *δ_i_*. We examined the fit of models with and without item–effect variables and investigated the hypothesized multidimensionality in our application. Although the model fit is equivalent for different identification schemes (e.g., for choosing a specific reference item), the disentangled information can differ. To account for this, we implemented different identification schemes. First, Item 3 “*I am satisfied with my life*” was used as the reference for scaling the latent states, as it is the most direct indicator of the construct of life satisfaction. Second, we chose Item 5 “*If I could live my life over, I would change almost nothing*” as the reference. As this item showed different psychometric properties in previous analysis (e.g., Pavot & Diener, 2008; Senecal et al., 2000) and it showed on average the largest difference in comparison to Item 3 in the application of Erhardt et al. (2022). Next to the multidimensionality itself, we investigated the incremental effect of the item–effect variables *δ_i_* as predictors of the two health outcomes (i.e., MHI-5 or PHI index) as compared with the latent state variables *η_t_* alone. To do so, we evaluated the incremental variance explained in the outcomes and the standardized regression coefficients. The analyses were implemented twice with the identical sample but different measurement periods (2008–2010 and 2011–2013) to investigate the stability of the results.

We estimated the different structural equation models using a maximum likelihood algorithm in *lavaan* version 0.6–9 (Rosseel, 2012) with *R* version 4.0.1 (R Core Team, 2021) for the complete sample (*N = 2,543*). For the samples with incomplete data (*N*_p1_ *= 5,549* and *N*_p2_* = 5,248*) full information maximum likelihood estimation (e.g., Graham, 2009) was applied. The model fit was examined with the *root mean square error of approximation* (RMSEA), *comparative fit index* (CFI), *Tucker–Lewis Index* (TLI), and *standardized root mean square residual* (SRMR). In line with conventional standards (e.g., Schermelleh-Engel et al., 2003), we viewed models with CFI ≥ .95, TLI ≥ .95, RMSEA ≤ .08, and SRMR ≤ .10 as “acceptable,” while models with CFI ≥ .97, TLI ≥ .97, RMSEA ≤ .05, or SRMR ≤ .05 were considered as “good” fitting. Model comparisons were based on differences in the *Akaike information criterion* (AIC; Akaike, 1974) and the *Bayesian information criterion* (BIC; Schwarz, 1978) for which lower values indicate a better fit. We also report the results of log-likelihood difference tests, although, these are not very informative in our large sample because of the excessive power to identify even trivial effects.

### Open Practices

The raw data and study material are available to the research community at https://lissdata.nl. Moreover, a detailed analysis code that allows for reproducing the reported findings is available in a public repository at https://osf.io/ekcqh/?view_only=7085df46f5494121b23eae9b3c28ace1.

## Results

### Person-Specific Item Effects in the SWLS

In accordance with the initial theoretical considerations for the item content and presentation, we investigated the measurement model for the SWLS with the two different reference items (3 or 5) in the two measurement periods.

A unidimensional model for the SWLS was not supported at the different measurement periods as indicated by RMESAs > .11 and CFIs/TLIs < .90 (see Table 2). Instead, the inclusion of person-specific item effects resulted in substantially improved model fits. The choice of the reference item does not affect model fit, as both identification schemes are equally valid. At the two measurement periods, the RMSEAs were .04 and .05, while the SRMRs, CFIs, and TLIs fell at .02, .99, and .98, respectively. Moreover, all model comparisons using the information criteria favored the models with item-effect variables. The results for the incomplete data were the same (see Supplemental Table S6).

**Table 2.** Model Fit and Model Comparison for the Different Measurement Models of the SWLS in the Complete Data (N = 2,543).

Model with	χ^2^(*df*)	RMSEA [90% CI]	SRMR	CFI	TLI	AIC	BIC
2008–2010							
Latent states	3,315(103)*	.111 [.108, .114]	**.060**	.894	.892	98,838	99,024
+ item effects	479(85) *	**.043** [.039, .046]	**.019**	**.987**	**.984**	**96,038**	**96,330**
2011–2013							
Latent states	3,374(103)*	.118 [.115, .122]	**.057**	.887	.885	98,378	98,565
+ item effects	584(85)*	**.048** [.044, .052]	**.017**	**.985**	**.981**	**95,224**	**95,516**

*Note.* Printed in bold are model fit parameters that indicate a good/acceptable model fit (*RMSEA* ≤ .05 / .08; *CFI* ≥ .97 / .95 ; *TLI* ≥ .97 / .95, *SRMR* ≤ .05 / .10) and the smallest AIC and BIC in the model comparison (see Schermelleh-Engel et al., 2003). CI = confidence interval; RMSEA = root mean square error of approximation; SRMR = standardized root mean square residual; CFI = comparative fit index; TLI = Tucker–Lewis index; AIC = Akaike information criterion; BIC = Bayesian information criterion.

* *p* < .05.

Accordingly, we further investigated the parameter estimates of the multi-state model with item-effect variables. Table 3 provides the parameter estimates when defining the states with Reference Item 3 and the item-effect variables as stable intra-individual differences of each other item to this reference. The latent states show that the participants were, on average, rather satisfied with their lives with latent means around 5.5 on a 7-point scale. Substantial interindividual variation of around one scale point showed that respondents differed in their reported life satisfaction. Moreover, life satisfaction was a rather stable construct in the studied sample as demonstrated by the substantial correlation between the latent states that exceeded *r* = .75. Yet, as the means and standard deviations of the item–effect variables differed significantly from zero (*p* < .05), the person-specific item effects indicated that the SWLS cannot be considered a unidimensional measure. All means of the item–effect variables were negative showing that participants scored, on average, lower on other items than on the Reference Item 3. This seems plausible given that the reference item is the most general and less specific one as compared with the other four items. For Item 5, we obtained the largest mean difference to the reference item with nearly one scale point deviation. Also, substantial inter-individual variation is present for the person-specific item effects of Item 5, that is nearly as large as the variation in the states. The means and standard deviations of all other item–effect variables were smaller, but substantial inter-individual variations of at least 0.40 scale points on average were observed. The item–effect variables carried additional, largely independent information as compared with the state variables. The correlations between the states and the item–effect variables did not exceed |*r*| = .20. Moreover, we found a medium to high correlation between the item–effect variables of Items 1 and 2 as well as Items 4 and 5 because the item content of these item pairs seemed related. As such, the investigation of item–effect variables indicated some kind of semantic multidimensionality in relation to the item content or potentially also item-specific response styles or general person characteristics that may explain more similar responses in specific items. Importantly, comparable conclusions were derived at both measurement periods (see Table 3), suggesting that the observed results were robust across the observational periods. Also, parameter estimates from the incomplete samples were rather similar (see Supplemental Table S7).

**Table 3.** Means, Standard Deviations, and Correlations for the Latent State and Item–Effect Variables With Reference Item 3 in the Complete Data (N = 2,543).

Variable	*M*	*SD*	*η*_1_	*η*_2_	*η*_3_	*δ*_1_	*δ*_2_	*δ*_4_	*δ*_5_
2008–2010									
*η*_1_	5.565	0.977	1	.754	.698	**.021**	.066	−.135	**.002**
*η*_2_	5.548	0.977		1	.785	**.030**	**.035**	−.199	−**.018**
*η*_3_	5.516	0.997			1	.069	.085	−.200	−**.014**
*δ*_1_	−0.461	0.389				1	.626	.317	.316
*δ*_2_	−0.330	0.400					1	.227	.111
*δ*_4_	−0.277	0.562						1	.457
*δ*_5_	−0.990	0.889							1
2011–2013									
*η*_1_	5.505	0.997	1	.763	.737	**.062**	.082	−.161	**.005**
*η*_2_	5.500	0.998		1	.786	.078	.103	−.156	**.015**
*η*_3_	5.484	1.032			1	**.041**	.103	−.191	**.008**
*δ*_1_	−0.460	0.436				1	.691	.254	.331
*δ*_2_	−0.320	0.406					1	.131	.143
*δ*_4_	−0.214	0.532						1	.431
*δ*_5_	−0.898	0.902							1

*Note.* *η_t_* = Latent state variable measured by Item 3 at the measurement occasions *t* ∈ {1, 2, 3}; *δ_i_* = Latent item–effect variables for Item *i* ∈ {1, 2, 4, 5}. All means, standard deviations and most of the correlations were significantly different from zero at *p* < .05. Non-significant correlations are printed in bold.

Table 4 provides the parameter estimates when defining the states with Reference Item 5 and the item-effect variables representing the inter-individual differences in the responses to each item as compared with Item 5. Rating life satisfaction with Item 5, as one would change almost nothing in life results in lower means of the states of around 4.5 (i.e., around one scale point lower), but larger inter-individual differences (i.e., *SD* of around 1.3 scale points). The stability of the states, now defined with Reference Item 5, is even larger as indicated by correlations that exceed *r* = .80. Again, all item–effect variables have means and standard deviations that differed significantly from zero (*p* < .05), such that SWLS cannot be considered a unidimensional measure. Yet, the direction, size, as well as correlation structure of the item–effect variables is different, due to the change in the reference item. The means of all item–effect variables were positive, showing that participants scored, on average, higher on other items than on the Reference Item 5, with the largest difference in comparison to Item 3. The standard deviations of all item-effect variables were around 0.8 scale points. With this identification scheme, the item–effect variables were medium to highly correlated with the states. Thus, the degree to which one would change almost nothing in life is substantially related to a systematically different responding on other items—underscoring that the content or response style regarding Item 5 is in part different from the other items. All item–effect variables are highly related to each other (i.e., correlations exceed *r* = .75). Again, the observed results were robust across the observational periods (see Table 4) and the sample specification (see Supplemental Table S8).

**Table 4:** Means, Standard Deviations, and Correlations for the Latent State and Item–Effect Variables With Reference Item 5 in the Complete Data (N = 2,543).

Variable	*M*	*SD*	η_*1*_	η_*2*_	η_*3*_	δ_*1*_	δ_*2*_	δ_*3*_	δ_*4*_
2008–2010									
η_*1*_	4.576	1.322	1	.865	.832	−.601	−.589	−.674	−.599
η_*2*_	4.558	1.309		1	.880	−.588	−.590	−.666	−.622
η_*3*_	4.527	1.327			1	−.569	−.569	−.660	−.619
δ_*1*_	0.529	0.850				1	.931	.901	.762
δ_*2*_	0.660	0.934					1	.905	.762
δ_*3*_	0.990	0.889						1	.784
δ_*4*_	0.712	0.806							1
2011–2013									
η_*1*_	4.607	1.348	1	.871	.854	−.569	−.581	−.673	−.625
η_*2*_	4.602	1.355		1	.882	−.568	−.578	−.677	−.628
η_*3*_	4.586	1.376			1	−.567	−.564	−.661	−.633
δ_*1*_	0.438	0.862				1	.935	.879	.752
δ_*2*_	0.577	0.935					1	.903	.754
δ_*3*_	0.898	0.902						1	.814
δ_*4*_	0.683	0.826							1

*Note.* *η_t_* = Latent state variable measured by Item 5 at the measurement occasions *t* ∈ {1, 2, 3}; *δ_i_* = Latent item–effect variables for Item *i* ∈ {1, 2, 3, 4}. All means, standard deviations, and correlations were significantly different from zero at *p* < .05.

### Prediction of Health Outcomes

To evaluate the relevance of the modeled item–effect variables for the prediction of the two health outcomes, we compared linear regressions of the MHI-5 or PHI index on either the latent state variables alone or on the latent state and item-effect variables together. Superior prediction accuracy of the latter would indicate that item-effect variables contain substantive information for the prediction of health. The respective regression results are summarized in Table 5 for the different outcomes, measurement periods, and identification schemes. Detailed results on model fit comparisons for the analysis without and with item-effect variables are provided in Table S5 in the Supplemental Material.

**Table 5:** Results of Linear Regressions of Health Outcomes in the Complete Data (N = 2,543).

Predictor	MHI				PHI			
	M1	M2	M3	M4	M1	M2	M3	M4
2008–2010								
η_*1*_	−.065*	−.063	.062*	−.085	−.151*	−.120*	−.097	−.162*
η_*2*_	−.198*	−.196*	−.196*	−.263*	−.074	−.100	−.047	−.134
η_*3*_	−.314*	−.292*	−.323*	−.388*	−.157*	−.101*	−.138*	−.135*
δ_*1*_		.022		.048		.158*		.344*
δ_*2*_		−.083*		−.193*		−.359*		−.836*
δ_*3*_		ref		−.436*		ref		.037
δ_*4*_		.084*		.120*		.108*		.247*
δ_*5*_		−.058*		ref		−.062		ref
*R* ^2^	.288	.292	.203	.292	.122	.192	.072	.192
Δ*R*^2^	.004		.089		.089		.120	
2011–2013								
η_*1*_	−.115*	−.110*	−.043	−.149*	−.193*	−.188*	−.186*	−.254*
η_*2*_	−.107*	−.097*	−.019	−.132*	−.022	−.004	−.050	−.005
η_*3*_	−.381*	−.377*	−.443*	−.503*	−.139*	−.111*	−.140*	−.148*
δ_*1*_		−.072		−.142		.052		.102
δ_*2*_		.018		.041		−.266*		−.611*
δ_*3*_		ref		−.437*		ref		.209
δ_*4*_		.051		−.080		.002		.003
δ_*5*_		−.059*		ref		−.000		ref
*R* ^2^	.318	.321	.247	.321	.108	.148	.072	.148
Δ*R*^2^	.003		.074		.040		.076	

*Note.* Standardized regression coefficients of four different models: M1 and M3 include *η_t_* = latent state variables measured by the reference item at the measurement occasions *t* ∈ {1, 2, 3}. M2 and M4 add *δ_i_* = latent item–effect variables for Item *i* ∈ {1, 2, 3, 4, 5} except for the reference item (ref) that is Item 3 in M1 and M2 or Item 5 in M3 and M4. *regression coefficients were significantly different from zero at *p* < .05. *R*^2^ *=* explained variance by the respective model on the outcome MHI or PHI, and *ΔR*^2^ = difference in *R*^2^. MHI = Mental Health Inventory; PHI = Physical Health/Mobility Index.

The choice of the reference item had no impact on the explained variance in the complete model using states and item–effect variables as predictors because both identification schemes are equally valid. Thereby, the latent variables of the SWLS scale could explain substantial variance in the MHI-5 index with slight differences between the measurement periods (2010 = 29.9% explained variance, 2013 = 32.1% explained variance) as well as in the PHI index (2010 = 19.2% explained variance, 2013 = 14.8% explained variance). As such, life satisfaction measures that account for item–effect variables, stronger predict the mental health index than the physical health index. The relevance of item–effect variables in this prediction substantially depended on the used identification scheme.

For the MHI-5 index, the latent states (i.e., life satisfaction measured by Reference Item 3) had a substantial impact and explained around 30% of the variance. As expected, higher life satisfaction indicated lower psychological distress. Although the most recent measurement of life satisfaction exhibited the strongest effect, the previous state variables added incremental information. This was not the case for the item–effect variables which increased the explained variance by <0.5%. A similar pattern was found for both measurement periods. As such, we can consider the item–effect variables as a nuisance without substantial meaning in this analysis. In contrast, the latent states (i.e., life satisfaction measured by the Reference Item 5) had a less strong impact and explained only around 20% of the variance, whereas again the most recent measure exhibited the strongest effect. In this case, substantial incremental information was added by the item–effect variables, whereas the specific effect of Item 3 was the most important. These two analyses suggest that primarily Item 3 substantially predicted the MHI-5 index.

A different pattern emerged for the PHI index. Life satisfaction measured by Reference Item 3 was less relevant for predicting physical health problems and explained only around 12% of the outcome variance. Moreover, the first state variable had a comparable impact on the outcome as the most recent measurement. In addition, the impact of the state variables decreased when adding the item–effect variables as additional predictors which explained around 7% incremental variance. Thereby the effect of Item 2 “The conditions of my life are excellent” was most important for predicting physical health problems. Thus, fewer mobility problems are reported, especially if the participants have higher values on this item in comparison to the reference item that assesses general life satisfaction. This pattern was even more prominent when choosing Item 5 as the reference. The more specific specification of the states explained less variance of around 7%, and accordingly more incremental variance referred to the item–effect variables. Again, the item–effect variable of Item 2 was the strongest. Accordingly, the more nuanced construct specification offered more detailed insights into the psychological phenomenon of interest. Thereby a global specification of life satisfaction was good for explaining mental health, but a more concrete specification based on the conditions of life was beneficial for predicting physical health. The overall pattern of the results was comparable in both measurement periods (see Table 5). Also, the same differences between the two identification schemes, and the same results on the relevance of specific predictors can be obtained in the incomplete data. Yet, the explained variance was slightly lower in the larger samples (see Supplemental Table S9 for details). Overall, we can consider our results on the multidimensionality in the SWLS and their predictive validity as stable, at least in our large sample.

## Discussion

Measurement models with person-specific item effects can contribute to psychological assessment by revealing multidimensionality in item responses and identifying secondary content traits with substantial meaning beyond the primary trait. We showed this by modeling a well-studied instrument (i.e., SWLS) as a multidimensional construct. In contrast to previous research that primarily viewed person-specific item effects as an unwanted source of nuisance (e.g., Eid & Kutscher, 2014; Joshanloo, 2022; López-Benítez et al., 2019; Scarpato et al., 2021), the present study adopted an alternative stance and considered them a meaningful subject of investigation. Specifically, in relation to theoretical considerations and previous investigations on the meaning of person-specific item effects in the SWLS, we pointed out how such effects can be informative for substantive analyses. Thereby, we showed how to separate systematic variance components that are common for all items of a scale from measurement error-free and stable item–effect variables using well-defined latent variables in the tradition of LST-R theory (Pohl et al., 2008; Steyer et al., 2015). This approach allowed for closely studying item–effect variables themselves and for using them in subsequent analysis. Importantly, we showed the predictive validity of item–effect variables that is plausible in relation to the item content. Our results suggest that responses to the SWLS are more stronger related to an indicator of mental health than of physical health (i.e., the latent variables of all items together explained up to 30% or 20% of the respective health index). This general result on the predictive validity is supported by previous studies, which investigated comparable constructs in large community samples, but modeled the SWLS as a unified factor (e.g., Cheung & Lucas, 2014; Hinz et al., 2018). In addition, we showed that a general definition of life satisfaction (i.e., in terms of Item 3 “I am satisfied with my life”) was sufficient for investigating the relation with mental health, but a more differentiated view was beneficial for predicting physical health. Especially person-specific effects for Item 2 “The conditions of my life are excellent” contributed to the explanation of physical health next to a general construct definition. This might support the interpretation of person-specific item effects as secondary traits with substantial meaning (similar to so-called personality nuances; e.g., McCrae et al., 2019; Stewart et al., 2021).

### Implications for Psychological Assessment

The study of person-specific item effects can support applied psychological assessments in several ways. As has been demonstrated in our application, the inclusion of person-specific item effects can guard against severe misspecifications of measurement models. This does not only improve conventional indices of model fit but also prevents severe structural parameter bias in, for example, regression weights or explained variances (McClure et al., 2021; Rhemtulla et al., 2020). More importantly, when person-specific item effects are viewed as a secondary trait rather than mere measurement bias, they provide additional information on individual differences between respondents without requiring the administration of additional items. Consequently, modeling person-specific item effects allows for more parsimonious assessment instruments. Moreover, in contrast to previous research on the incremental contribution of single items for personality research (e.g., Achaa-Amankwaa et al., 2021; Stewart et al., 2021), our modeling approach managed to specify proper measurement models for each item effect. As such, systematic item–effect variables were distinguished from random measurement error and, thus, allowed examining true score effects with criterion variables. For such analysis, particularly the interpretation and specific source of person-specific item effects is important. To prevent from ad hoc secondary analyses without careful considering the conceptual questions, *a priori* theoretical underpinning is important—because the meaning of person-specific item effects is more ambiguous as compared with method effects that draw on specific item characteristics like wording, rater groups, or response styles (e.g., Eid, 2000; Henninger & Meiser, 2020a, 2020b; Kam & Fan, 2020; Koch et al., 2018; Pohl et al., 2008). Considerations should carefully examine possible semantic multidimensionality but also possible multidimensionality in relation to the item formats. Furthermore, the nomological net of item–effect variables (Erhardt et al., 2022) as well as their comparative predictive strengths can be evaluated to derive conceptual clarity for the identified latent variables. Finally, we highlighted how the chosen identifying constraints for the latent variables can affect the interpretation of the results. As has already been noted in other contexts (e.g., Eid et al., 2003; Little et al., 2006), the choice of the reference item might be arbitrary from a model fit perspective, but it is not for the interpretation of the resulting validity coefficients. We recommend a theory-guided justification for the choice of reference items in latent variable models. Note, in case of using the models for group comparisons, then no person-specific item effects should be prevalent for the reference item to allow for ensuring construct equivalence between subgroups (see e.g., Kopf et al., 2015 for the specification of anchor items in case of differential item functioning).

### Limitations and Directions for Future Research

The present study focused on the advantages of modeling person-specific item effects in the SWLS to strengthen the evidence on the impact of such effects for psychometric and substantive analyses. Accordingly, we emphasized its potential for applied psychological measurement and also provided the respective analysis code to aid similar analyses for future research. However, we readily acknowledge that the presentation of the technical details of our modeling approach in the Appendix was rather concise. A detailed psychometric introduction into modeling item–effect variables is given in Erhardt et al. (2022) and Thielemann et al. (2017), while the strengths of the LST-R theory, in general, are described, for instance, in Steyer et al. (2015), Geiser and Lockhart (2012), or Eid (1996). Moreover, the benefits of separating item–effect variables for investigating their source were recently also pointed out by van Bork et al. (2022). Furthermore, the presented model should be considered a starting point for future research. For example, one possible extension might be accounting for common method effects (e.g., Holtmann et al., 2020) or adjusting for explanatory variables (Thielemann et al., 2017) when investigating the validity of person-specific item effects. However, because the model is already rather complex, it remains to be seen whether these model extensions can be useful for applications on a broader scale. Another downside of the presented analyses with item–effect variables is their increased complexity in comparison to traditional unidimensional construct definitions in terms of (a) data requirements, (b) model specification, (c) model inspection, (d) subsequent analyses, and (e) scientific communication. For example, modeling item–effect variables in longitudinal data requires at least three measurement occasions for the same persons and items. More latent variables have to be identified with specific model assumptions on the stability of the person-specific item effects that might or might not be violated in a specific situation. If multidimensionality is observed (i.e., substantial inter-individual differences are prevalent on item–effect variables), it is not clear without ancillary information whether these represent trait-relevant item content or rather some form of measurement bias such as motivational characteristics or specific response styles. It can also be computational more demanding and more challenging to incorporate an item-based specification of a focal construct in substantial analyses; especially when the relations among multiple constructs are of interest. Finally, the requirements for a comprehensive reporting of respective results increase because various multivariate relations are possible and the choice of a specific identification scheme can substantially impact the disentangled information. Thus, even though the results on the person-specific item effects are promising in our application on the five items SWLS, whether these advantages outweigh the potential drawbacks needs to be answered for each application and setting anew.

### Conclusion

Recent advances in psychometric modeling allow in-depth evaluations of person-specific item effects beyond the common trait. The present study identified relevant item–effect variables in the SWLS and, more importantly, demonstrated their stability and incremental predictive validity. As such, we showed that the more nuanced construct definition in relation to the individual items could offer a much more detailed perspective for predicting mental and physical health outcomes. Although these modeling approaches require a profound psychometric understanding because they are substantially more complex as compared with traditional unidimensional construct definitions, we believe that item–effect variables are a promising path for future research that allow more nuanced construct specifications and more detailed insights into psychological phenomena.

## Supplemental Material

sj-docx-1-asm-10.1177_10731911221149949 – Supplemental material for The Predictive Validity of Item Effect Variables in the Satisfaction With Life Scale for Psychological and Physical Health Click here for additional data file. Supplemental material, sj-docx-1-asm-10.1177_10731911221149949 for The Predictive Validity of Item Effect Variables in the Satisfaction With Life Scale for Psychological and Physical Health by Marie-Ann Sengewald, Tina H. Erhardt and Timo Gnambs in Assessment

## Appendix: Modeling Person-Specific Item Effects

In general, method effects can be present when assessing latent constructs with multi-item scales (e.g., Campbell & Fiske, 1959; Lord & Novick, 1968; Steyer et al., 2015). In cross-sectional data, multitrait-multimethod (MTMM) models can be used to account for homogeneous method effects that generalize across, for example, different items of a scale, like differences between positive and negative item formulations, or between multiple rater groups (e.g., Eid, 2000; Henninger & Meiser, 2020a, 2020b; Kam & Fan, 2020; Koch et al., 2018; Pohl et al., 2008). This restrictive assumption has been relaxed in longitudinal data to acknowledge method effects for individual items (e.g., Cogo-Moreira et al., 2021; Eid, 1996; Eid & Kutscher, 2014; Erhardt et al., 2022; Geiser & Lockhart, 2012; Holtmann et al., 2020; Marsh & Grayson, 1994; Thielemann et al., 2017). In the following, we investigate the multidimensionality that is implied by this approach and describe how multi-state models can be extended for including item–effect variables that disentangle item-specific variance components.

### Multi-State Model

Figure 1 illustrates a basic multi-state model for three measurement occasions (left plot). First, following the (revised) LST-R theory (Steyer et al., 2015), the observed item responses *Y*_it_ are decomposed into a common latent state variable η_*t*_ at each time point *t* and the respective measurement error ε_*it*_ for each manifest indicator *i*. More precisely, *Y_it_* = λ_*it*0_ + λ_*it*1_η_*t*_ + ε_*it*_ item-specific intercept λ_*it*0_ and factor loadings λ_*it*1_ . For defining the scale of η_*t*_, a reference item can be chosen, by constraining its intercept to zero and its factor loading to one. However, other identification schemes are possible and equally valid (see, for example, Schroeders & Gnambs, 2020; Steyer et al., 2015). In addition, following Millsap (2011), we assume strong measurement invariance (i.e., identical factor loadings and intercepts) for all measurement occasions. Finally, the measurement error variables are uncorrelated with each other and the latent state variables (see Steyer et al., 2015). With these model assumptions, the common latent states η_*t*_ represent a unidimensional attribute of the persons in a specific situation at time *t* that is measured on an identical scale across all measurement occasions (i.e., on the scale of the reference item). The observed responses of the persons differ only due to random measurement error and item parameters that are constant for all persons. Thus, all systematic variation in the item responses is represented in the common latent states η_*t*_. Yet, the assumptions of a multi-state model may not hold in practice, especially, if person-specific item effects are present.

### Person-Specific Item Effects

Method effects can be defined as regression residuals (see Eid, 2000) or as latent differences (see Pohl et al., 2008). In line with LST-R theory, both approaches can be used for acknowledging method effects on the item level (e.g., Geiser & Lockhart, 2012; Thielemann et al., 2017). We extend the multi-state model for latent difference variables *δ_t_* (see the right plot in Figure 1) and point out differences to the residual definition. As in the multi-state model, the scale of the latent state variables η_*t*_ can be defined with a reference item. Then, the item effect variables δ_*i*_ describe interindividual differences, when using another item than the reference item for assessing the focal construct. For all items, the intercepts have to be set equal to zero and all loadings equal to one. Accordingly, the indicators are modeled as *Y_it_* = η_*t*_ + δ_*i*_ + ε_*it*_, except for the reference item that has no item–effect variable (i.e., the number of item- effect variables δ_*i*_ is one less than the number of items). Thus, instead of modeling item parameters that are constant for all persons, the person-specific item effects are modeled as additional variables that represent interindividual differences in responding to a specific item as compared with the reference item. The identification of the person-specific item effects requires longitudinal data on at least three measurement occasions and the assumption of identical individual item effects across measurement occasions, meaning that the item–effect variables are identical over time (see Figure 1B). For this reason, the time index *t* is omitted for δ_*i*_. In addition, measurement error variables have to be uncorrelated. With these model assumptions, the systematic variation in the item responses is represented in the common latent states η_*t*_ and the item effect variables δ_*i*_. Basically, the same differences can be modeled when person-specific item effects are specified as regression residuals (e.g., Cogo-Moreira et al., 2021; Geiser et al., 2019; Holtmann et al., 2020). Yet, the information is represented in different parameters and assumptions are made in each approach (e.g., residuals are typically specified with mean zero and item-specific intercepts, also residuals are commonly uncorrelated with latent states; see Geiser & Lockhart, 2012 for a detailed comparison). For our application, we use the definition as latent difference variables, because this representation is straightforward for subsequent multivariate analysis and symmetrical with respect to the selected reference method—so that the choice of the reference method does not affect the model fit (Pohl et al., 2008). However, the chosen identifying constraints for the latent variables can affect the interpretation of the latent variables (e.g., Eid et al., 2003; Little et al., 2006; Pohl et al., 2008). In general, the states represent the attribute measured by the reference item and the person-specific item effects represent the differences to this reference. However, it is important to note that also alternative identification constraints have been proposed. For example, one approach defines latent states as the average of all items and considers the deviation of the individual items from the average (i.e., relying on an effect coding instead of dummy coding strategy; see Pohl & Steyer, 2010; Thielemann et al., 2017). We choose the reference method as this allows for comparing the content of the reference item and every other item for investigating person-specific item effects. Consequently, the means, variances, and correlation coefficients of the latent variables depend on the chosen reference item.
