# Supplementary material for: The Predictive Validity of Item Effect Variables in the Satisfaction With Life Scale for Psychological and Physical Health
Source: Assessment. 2023 Feb 8;30(8):2461–75. doi: 10.1177/10731911221149949 (PMC10623622; doi:10.1177/10731911221149949)
Supplement: sj-docx-1-asm-10.1177_10731911221149949 – Supplemental material for The Predictive Validity of Item Effect Variables in the Satisfaction With Life Scale for Psychological and Physical Health [file sj-docx-1-asm-10.1177_10731911221149949.docx]

Supplemental Material for

**The Predictive Validity of** **Item Effect Variables in the *Satisfaction With Life Scale* for Psychological and Physical Health**

In the following, we provide details for the measures and model comparisons in our analysis. Moreover, a detailed analysis code is available at a public repository and can be achieved under <https://osf.io/ekcqh/?view_only=7085df46f5494121b23eae9b3c28ace1>.

Table S1 summarizes descriptive statistics for the five items of the *Satisfaction with Life Scale* (SWLS; Diener et al., 1985), showing that the ratings on the 7-point response scale substantially varied between the persons, and that on average life satisfaction was rather high.

Table S2 and S3 provide the scale description for the outcome measures, the *Mental Health Inventory* (MHI-5; Moor et al., 2018; Ostroff et al., 1996) and *Physical Health / Mobility Index* (PHI; Green et al., 2001), respectively. For each outcome, we created an index by averaging the available item responses on the respective instrument for each person. Descriptive statistics for the index at the different measurement occasions 2010 and 2013 are summarized in Table S4.

Table S5 summarizes results on information criteria and difference tests for the model comparisons on the predictive validity of item-specific method effects. For this we predicted the different outcomes in a linear regression model either only by latent state variables for life satisfaction, or by latent state variables and item-specific method effects. The analysis was considered for the two outcome measures, at the two measurement periods and with two identification schemes (i.e., using Item 3 or Item 5 of the SWLS as the reference item).

Finally, we summarize the results for the analysis with incomplete data. Table S6 presents the fit of the measurement models. Tables S7 and S8 show the results for the latent variables, respectively for reference Item 3 and 5. Then, Table S9 shows the results for the different prediction models. The same conclusions can be drawn as with the complete data.

**Table S1**

*Descriptive Statistics for the SWLS items* *at Six Measurement Occasions (2008 - 2013)*

|  | *Min* | *Max* | *M* | *SD* | skew | kurtosis |
| --- | --- | --- | --- | --- | --- | --- |
| cp08a014 | 1.00 | 7.00 | 5.11 | 1.19 | -1.16 | 1.60 |
| cp08a015 | 1.00 | 7.00 | 5.24 | 1.22 | -1.12 | 1.25 |
| cp08a016 | 1.00 | 7.00 | 5.57 | 1.08 | -1.45 | 2.78 |
| cp08a017 | 1.00 | 7.00 | 5.28 | 1.25 | -1.05 | 1.08 |
| cp08a018 | 1.00 | 7.00 | 4.56 | 1.61 | -0.50 | -0.65 |
| cp09b014 | 1.00 | 7.00 | 5.08 | 1.18 | -1.09 | 1.32 |
| cp09b015 | 1.00 | 7.00 | 5.23 | 1.20 | -1.07 | 1.14 |
| cp09b016 | 1.00 | 7.00 | 5.55 | 1.09 | -1.37 | 2.31 |
| cp09b017 | 1.00 | 7.00 | 5.26 | 1.23 | -1.06 | 1.14 |
| cp09b018 | 1.00 | 7.00 | 4.54 | 1.56 | -0.47 | -0.68 |
| cp10c014 | 1.00 | 7.00 | 5.05 | 1.23 | -1.11 | 1.26 |
| cp10c015 | 1.00 | 7.00 | 5.16 | 1.27 | -1.07 | 0.94 |
| cp10c016 | 1.00 | 7.00 | 5.52 | 1.10 | -1.34 | 2.23 |
| cp10c017 | 1.00 | 7.00 | 5.25 | 1.23 | -0.97 | 0.85 |
| cp10c018 | 1.00 | 7.00 | 4.57 | 1.58 | -0.51 | -0.56 |
| cp11d014 | 1.00 | 7.00 | 5.06 | 1.23 | -1.11 | 1.16 |
| cp11d015 | 1.00 | 7.00 | 5.21 | 1.22 | -1.08 | 1.10 |
| cp11d016 | 1.00 | 7.00 | 5.49 | 1.09 | -1.38 | 2.38 |
| cp11d017 | 1.00 | 7.00 | 5.26 | 1.22 | -1.05 | 1.01 |
| cp11d018 | 1.00 | 7.00 | 4.61 | 1.58 | -0.58 | -0.54 |
| cp12e014 | 1.00 | 7.00 | 5.04 | 1.25 | -1.10 | 1.10 |
| cp12e015 | 1.00 | 7.00 | 5.17 | 1.26 | -1.07 | 0.91 |
| cp12e016 | 1.00 | 7.00 | 5.50 | 1.10 | -1.40 | 2.48 |
| cp12e017 | 1.00 | 7.00 | 5.29 | 1.21 | -1.08 | 1.17 |
| cp12e018 | 1.00 | 7.00 | 4.61 | 1.59 | -0.53 | -0.61 |
| cp13f014 | 1.00 | 7.00 | 5.01 | 1.30 | -1.07 | 0.93 |
| cp13f015 | 1.00 | 7.00 | 5.14 | 1.30 | -1.05 | 0.81 |
| cp13f016 | 1.00 | 7.00 | 5.50 | 1.14 | -1.36 | 2.17 |
| cp13f017 | 1.00 | 7.00 | 5.29 | 1.23 | -1.07 | 1.22 |
| cp13f018 | 1.00 | 7.00 | 4.57 | 1.63 | -0.52 | -0.69 |

**Table S2**

*Mental Health Inventory (MHI-5; Moore et al., 2018)*

| The following questions are about how you felt over the past month. For every question, please choose the answer that best describes how you felt during this past month.  This past month .... | |
| --- | --- |
| 011 | I felt very anxious |
| 012 | I felt so down that nothing could cheer me up |
| 013 | *I felt calm and peaceful^*^* |
| 014 | I felt depressed and gloomy |
| 015 | *I felt happy^*^* |
| Response scale: 1 = *never*, 2 = *seldom*, 3 = *sometimes*, 4 = *often*, 5 = *mostly*, 6 = *continuously*  *^*^reverse coded* | |

**Table S3**

*Physical Health / Mobility Index (PHI; Green et al., 2001)*

| Below you will find a number of actions that some people have difficulties with. Can you indicate, for each activity, whether you can perform it without any trouble, with some trouble, with a lot of trouble, only with the help of others or not at all?  This question does not apply to problems which you expect will not last longer than three months. | |
| --- | --- |
| 023 | walking 100 meters |
| 024 | sitting for around two hours |
| 025 | getting up from a chair in which you sat for some time |
| 026 | walking several stairs without resting in between |
| 027 | walking up a staircase without resting |
| 028 | crouching, kneeling, crawling on all fours |
| 029 | reaching above shoulder height or stretching your arms above shoulder height |
| 030 | moving large objects such as a dining room chair |
| 031 | lifting or carrying a weight of 5 kilos, such as a heavy bag of groceries |
| 032 | picking up a small coin lying on the table |
| 033 | dressing and undressing, including shoes and socks |
| 034 | walking across the room |
| 035 | bathing or showering |
| 036 | eating, such as cutting your food into small bits |
| 037 | getting in and out of bed |
| 038 | using the toilet, including sitting down and standing up |
| 039 | reading a map to find your way in an unfamiliar area |
| 040 | preparing a hot meal |
| 041 | shopping |
| 042 | telephoning |
| 043 | taking medicines |
| 044 | performing housekeeping work or maintaining the garden |
| 045 | taking care of financial affairs, such as paying bills and keeping track of expenditure |
| Response scale: 1 = *without any trouble*, 2 = *with some trouble*, 3 = *with a lot of trouble*, 4 = *only with the help of others*, 5 = *not at all* | |

**Table S4**

*Descriptive Statistics for the MHI and PHI Scores in 2010 and 2013*

| Year | *Min* | *Max* | *M* | *SD* | skew | kurtosis |
| --- | --- | --- | --- | --- | --- | --- |
| MHI |  |  |  |  |  |  |
| $\mathbf{2010}$ | 1.00 | 6.00 | 2.21 | 0.82 | 1.17 | 1.36 |
| $\mathbf{2013}$ | 1.00 | 6.00 | 2.17 | 0.82 | 1.08 | 1.09 |
| PHI |  |  |  |  |  |  |
| 2010 | 1.00 | 3.70 | 1.21 | 0.34 | 2.75 | 9.18 |
| 2013 | 1.00 | 5.00 | 1.24 | 0.38 | 2.79 | 10.70 |

**Table S5**

*Results on Information Criteria and Difference Tests*

| Outcome   \| Predictors \| \| --- \| | AIC | BIC | $\Delta\chi^{2}\left( df \right)=p$ |
| --- | --- | --- | --- | --- |
| MHI 2010 |  |  |  |
| latent states (3) | 101453 | **101775** | 14.70(4) < .01 |
| + item effects (3) | **101447** | 101791 |  |
| latent states (5) | 101765 | 102086 | 325.99(4) < .001 |
| + item effects (5) | **101447** | **101791** |  |
| MHI 2013 |  |  |  |
| latent states (3) | 100536 | **100858** | 16.49(4) < .01 |
| + item effects (3) | **100528** | 100873 |  |
| latent states (5) | 100823 | 101145 | 303.50(4) < .001 |
| + item effects (5) | **100528** | **100873** |  |
| PHI 2010 |  |  |  |
| latent states (3) | 97469 | 97790 | 104.27(4) < .001 |
| + item effects (3) | **97372** | **97717** |  |
| latent states (5) | 97612 | 97934 | 248.03(4) < 0.001 |
| + item effects (5) | **97372** | **97717** |  |
| PHI 2013 |  |  |  |
| latent states (3) | 97278 | 97600 | 73.33(4) < .001 |
| + item effects (3) | **97213** | **97558** |  |
| latent states (5) | 97383 | 97704 | 177.65(4) < .001 |
| + item effects (5) | **97213** | **97558** |  |

*Note.* Printed in bold are the smallest AIC and BIC in the respective model comparison without and with item effects for the different outcomes at different measurement occasions and the different identification schemes using either Item 3 or Item 5 for defining the latent states and item-effect variables.

**Table S6**

*Model Fit and Model Comparison for the Different Measurement Models of the SWLS in the Incomplete Data (N = 5,549 or N = 5,248 for the first or second measurement period).*

| Model with | | $\chi^{2}(df)$ | RMSEA [90% CI] | SRMR | CFI | TLI | AIC | BIC |
| --- | --- | --- | --- | --- | --- | --- | --- | --- |
| 2008-2010 | latent states | 5088(103)* | .093 [.091; .096] | **.059** | .904 | .902 | 181383 | 181594 |
|  | + item effects | 731(85)* | **.037** [.035; .040] | **.017** | **.988** | **.985** | **177061** | **177392** |
| 2011-2013 | latent states | 5752(103)* | .102 [.100; .104] | **.059** | .895 | .893 | 179426 | 179636 |
|  | + item effects | 813(85)* | **.040** [.038; .043] | **.017** | **.987** | **.983** | **174523** | **174851** |

*Note.* RMSEA = Root mean square error of approximation, SRMR = Standardized root mean square residual, CFI = Comparative fit index, TLI = Tucker-Lewis index, AIC = Akaike information criterion, BIC = Bayesian information criterion, CI = confidence interval. Printed in bold are model fit parameters that indicate a good/acceptable model fit ($RMSEA\leq.05/.08$; $CFI\geq.97/.95$; $TLI\geq.97/.95, SRMR\leq.05/.10$) and the smallest AIC and BIC in the model comparison (see Schermelleh-Engel et al., 2003). * *p* < .05.

**Table S7**

*Means, Standard Deviations, and Correlations for the Latent State and Item-Effect Variables with Reference Item 3 in the Incomplete Data (N = 5,549 or N = 5,248 for the first or second measurement period).*

| Variable | | *M* | *SD* | $\eta_{1}$ | $\eta_{2}$ | $\eta_{3}$ | $\delta_{1}$ | $\delta_{2}$ | $\delta_{4}$ | $\delta_{5}$ |
| --- | --- | --- | --- | --- | --- | --- | --- | --- | --- | --- |
| 2008 - 2010 | $\eta_{1}$ | 5.517 | 0.983 | 1 | .724 | .687 | **.026** | **.032** | -.143 | **-.008** |
|  | $\eta_{2}$ | 5.496 | 1.012 |  | 1 | .799 | .051 | .047 | -.160 | **-.028** |
|  | $\eta_{3}$ | 5.452 | 1.023 |  |  | 1 | .050 | .073 | -.188 | -.048 |
|  | $\delta_{1}$ | -0.450 | 0.392 |  |  |  | 1 | .623 | .292 | .300 |
|  | $\delta_{2}$ | -0.330 | 0.429 |  |  |  |  | 1 | .179 | .115 |
|  | $\delta_{4}$ | -0.280 | 0.565 |  |  |  |  |  | 1 | .466 |
|  | $\delta_{5}$ | -0.978 | 0.894 |  |  |  |  |  |  | 1 |
| 2011 - 2013 | $\eta_{1}$ | 5.471 | 1.001 | 1 | .752 | .720 | .050 | .057 | -.146 | **-.003** |
|  | $\eta_{2}$ | 5.465 | 1.009 |  | 1 | .763 | .068 | .071 | -.150 | **-.001** |
|  | $\eta_{3}$ | 5.443 | 1.039 |  |  | 1 | **.019** | .062 | -.173 | **-.010** |
|  | $\delta_{1}$ | -0.458 | 0.418 |  |  |  | 1 | .656 | .214 | .262 |
|  | $\delta_{2}$ | -0.331 | 0.420 |  |  |  |  | 1 | .124 | .100 |
|  | $\delta_{4}$ | -0.241 | 0.548 |  |  |  |  |  | 1 | .435 |
|  | $\delta_{5}$ | -0.910 | 0.898 |  |  |  |  |  |  | 1 |

*Note.* $\eta_{t}$ = Latent state variable measured by item 3 at the measurement occasions $t\in\left\{ 1,2,3 \right\}$; $\delta_{i}$ = Latent item-effect variables for item $i\in\left\{ 1,2,4,5 \right\}$. All means, standard deviations and most of the correlations were significantly different from zero at *p* < .05. Non-significant correlations are printed in bold.

**Table S8**

*Means, Standard Deviations, and Correlations for the Latent State and Item-Effect Variables with Reference Item 5 in the Incomplete Data (N = 5,549 and N = 5,248 for the first or second measurement period).*

| Variable | | *M* | *SD* | $\eta_{1}$ | $\eta_{2}$ | $\eta_{3}$ | $\delta_{1}$ | $\delta_{2}$ | $\delta_{3}$ | $\delta_{4}$ |
| --- | --- | --- | --- | --- | --- | --- | --- | --- | --- | --- |
| 2008 – 2010 | $\eta_{1}$ | 4.540 | 1.324 | 1 | .846 | .818 | -.593 | -.587 | -.669 | -.597 |
|  | $\eta_{2}$ | 4.518 | 1.332 |  | 1 | .866 | -.565 | -.563 | -.650 | -.588 |
|  | $\eta_{3}$ | 4.474 | 1.325 |  |  | 1 | -.551 | -.541 | -.637 | -.589 |
|  | $\delta_{1}$ | 0.528 | 0.861 |  |  |  | 1 | .926 | .900 | .754 |
|  | $\delta_{2}$ | 0.648 | 0.946 |  |  |  |  | 1 | .893 | .740 |
|  | $\delta_{3}$ | 0.978 | 0.894 |  |  |  |  |  | 1 | .784 |
|  | $\delta_{4}$ | 0.698 | 0.805 |  |  |  |  |  |  | 1 |
| 2011 – 2013 | $\eta_{1}$ | 4.561 | 1.342 | 1 | .862 | .841 | -.575 | -.580 | -.666 | -.605 |
|  | $\eta_{2}$ | 4.555 | 1.350 |  | 1 | .865 | -.567 | -.574 | -.664 | -.606 |
|  | $\eta_{3}$ | 4.533 | 1.367 |  |  | 1 | -.570 | -.563 | -.649 | -.605 |
|  | $\delta_{1}$ | 0.452 | 0.886 |  |  |  | 1 | .931 | .890 | .744 |
|  | $\delta_{2}$ | 0.579 | 0.952 |  |  |  |  | 1 | .743 | .899 |
|  | $\delta_{3}$ | 0.910 | 0.900 |  |  |  |  |  | 1 | .801 |
|  | $\delta_{4}$ | 0.669 | 0.823 |  |  |  |  |  |  | 1 |

*Note.* $\eta_{t}$ = Latent state variable measured by item 5 at the measurement occasions $t\in\left\{ 1,2,3 \right\}$; $\delta_{i}$ = Latent item-effect variables for item $i\in\left\{ 1,2,3,4 \right\}$. All means, standard deviations and correlations were significantly different from zero at *p* < .05.

**Table S9**

*Results of Linear Regressions of Health Outcomes in the Incomplete Data (N = 5,549 and N = 5,248 for the first or second measurement period).*

|  | | | MHI | | | | PHI | | | |
| --- | --- | --- | --- | --- | --- | --- | --- | --- | --- | --- |
| Predictor | | | M1 | M2 | M3 | M4 | M1 | M2 | M3 | M4 |
| 2008 – 2010 | $\eta_{1}$ | -.109* | | -.106* | .055 | -.143* | -.086* | -.084* | -.032 | -.162* |
|  | $\eta_{2}$ | -.180* | | -.180* | -.152* | -.263* | -.121* | -.126* | -.094* | -.134 |
|  | $\eta_{3}$ | -.275* | | -.261* | -.349* | -.388* | -.124* | -.073* | -.170* | -.135* |
|  | $\delta_{1}$ |  | | .023 |  | .049 |  | .144* |  | .344* |
|  | $\delta_{2}$ |  | | -.063* |  | -.138* |  | -.324* |  | -.836* |
|  | $\delta_{3}$ |  | | ref |  | -.466* |  | ref |  | .037 |
|  | $\delta_{4}$ |  | | .066* |  | .094* |  | .109* |  | 0.247* |
|  | $\delta_{5}$ |  | | -.041* |  | ref |  | -.042 |  | ref |
| $R^{2}$ |  | .265 | | .269 | .194 | .269 | .090 | .154 | .052 | .154 |
| $\Delta R^{2}$ |  | .004 | | | .075 | | .064 | | .102 | |
| 2011 – 2013 | $\eta_{1}$ | -.096* | | -.092* | .028 | -.123* | -.137* | -.130* | -.098* | -.174* |
|  | $\eta_{2}$ | -.130* | | -.124* | -.090* | -.166* | -.049 | -.037 | -.017 | -.050 |
|  | $\eta_{3}$ | -.353* | | -.347* | -.417* | -.457* | -.109* | -.093* | -.104* | -.122* |
|  | $\delta_{1}$ |  | | -.027 |  | -.058 |  | .054 |  | .114 |
|  | $\delta_{2}$ |  | | .004 |  | .010 |  | -.223* |  | -.506* |
|  | $\delta_{3}$ |  | | ref |  | -.497* |  | ref |  | .103 |
|  | $\delta_{4}$ |  | | .072* |  | -.109* |  | .015 |  | .022 |
|  | $\delta_{5}$ |  | | -.065* |  | ref |  | -.005 |  | ref |
| $R^{2}$ |  | .288 | | . 292 | .223 | .292 | .073 | .100 | .044 | .100 |
| $\Delta R^{2}$ |  | .004 | | | .069 | | .027 | | .056 | |

*Note.* Standardized regression coefficients of four different models: M1 and M3 include $\eta_{t}$ = latent state variables measured by the reference item at the measurement occasions $t\in\left\{ 1,2,3 \right\}$. M2 and M4 add $\delta_{i}$ = latent item-effect variables for item $i\in\left\{ 1,2,3,4,5 \right\}$ except for the reference item (ref) that is Item 3 in M1 and M2 or Item 5 in M3 and M4. * regression coefficients were significantly different from zero at *p* < .05. $R^{2}$ *=* explained variance by the respective model on the outcome MHI or PHI, and $\Delta R^{2}=$ difference in $R^{2}$.
